# Palliative Care in High-Grade Glioma: A Review

**DOI:** 10.3390/brainsci10100723

**Published:** 2020-10-13

**Authors:** Rita C. Crooms, Nathan E. Goldstein, Eli L. Diamond, Barbara G. Vickrey

**Affiliations:** 1 Icahn School of Medicine at Mount Sinai, 1 Gustave L. Levy Pl, New York, NY 10029, USA; nathan.goldstein@mssm.edu (N.E.G.); barbara.vickrey@mssm.edu (B.G.V.); 2 Geriatric Research Education and Clinical Center, James J. Peters VA Medical Center, 130 Kingsbridge Avenue, Bronx, NY 10468, USA; 3 Department of Neurology, Memorial Sloan Kettering Cancer Center, 1275 York Avenue, New York, NY 10065, USA; diamone1@mskcc.org

**Keywords:** palliative care, supportive care, glioma, quality of life

## Abstract

High-grade glioma (HGG) is characterized by debilitating neurologic symptoms and poor prognosis. Some of the suffering this disease engenders may be ameliorated through palliative care, which improves quality of life for seriously ill patients by optimizing symptom management and psychosocial support, which can be delivered concurrently with cancer-directed treatments. In this article, we review palliative care needs associated with HGG and identify opportunities for primary and specialty palliative care interventions. Patients with HGG and their caregivers experience high levels of distress due to physical, emotional, and cognitive symptoms that negatively impact quality of life and functional independence, all in the context of limited life expectancy. However, patients typically have limited contact with specialty palliative care until the end of life, and there is no established model for ensuring their palliative care needs are met throughout the disease course. We identify low rates of advance care planning, misconceptions about palliative care being synonymous with end-of-life care, and the unique neurologic needs of this patient population as some of the potential barriers to increased palliative interventions. Further research is needed to define the optimal roles of neuro-oncologists and palliative care specialists in the management of this illness and to establish appropriate timing and models for palliative care delivery.

## 1. Introduction

Approximately 17,000 new cases of high-grade glioma (HGG) are diagnosed in the United States each year. Though rare, this group of WHO Grade III and IV primary malignant brain tumors, including anaplastic oligodendroglioma, anaplastic astrocytoma, and glioblastoma, have a substantial impact: primary malignant brain tumors account for approximately 3% of all cancer deaths, the majority (around 80%) of which are HGG [1,2]. It remains incurable, with the median survival ranging from months to a few years depending on histologic, clinical, and molecular factors. HGG also carries a significant economic burden, with median direct cost per patient of $184,000 [3], which is higher than for other types of cancer (e.g., the median direct cost in lung cancer is ~$159,000 per patient) [4]. During the short course of their illness, patients have high symptom burden and supportive care needs that are distinct from those of other cancer patients [5]. The addition of palliative care to the neuro-oncology treatment model has the potential to address these needs.

Palliative care is an interdisciplinary medical specialty dedicated to relieving the symptoms and stress associated with serious illness, with the goal of improving quality of life for patients and caregivers. [6] It is appropriate at any stage of serious illness, regardless of prognosis, and can be provided concurrently with cancer-directed therapy. Palliative care has been demonstrated to be beneficial in terms of quality of life, patient and caregiver satisfaction, and health care costs [7,8,9,10,11]. The American Society of Clinical Oncology (ASCO) recommends that early palliative care be integrated into cancer treatment plans, yet there is no single palliative care delivery model that can be applied across all types of cancer [12]. HGG, in particular, has been recognized as unique in terms of patients’ palliative care needs. [13].

This review explores unmet needs and opportunities for palliative care for HGG patients and caregivers. We summarize current literature in the domains of (1) supportive care needs with respect to physical and emotional symptoms, functional impairments, and health-related quality of life (HRQOL), (2) caregiver needs, (3) advance care planning, (4) the end of life, and (5) utilization of primary and specialty palliative care in HGG. We further discuss possible barriers to implementation of palliative care in neuro-oncology and propose opportunities to improve research, education, and comprehensive patient care.

## 2. Approach

This is a narrative review intended to describe and discuss the use of palliative care in treating patients with high-grade glioma. We conducted a MEDLINE search using the terms ‘palliative care’ or ‘supportive care’ and ‘high-grade glioma’, ‘glioma’, or ‘malignant brain tumor’ in the title and/or abstract from 2000 through December 2019. Articles were included if the review subject or study population was patients with high-grade glioma or if there was subgroup analysis specific to high-grade glioma, and if they addressed one of the following topics: Physical, emotional, or cognitive symptoms; functional status; health-related quality of life; advance care planning; palliative care; or end-of-life and hospice. Articles were excluded if they did not specifically discuss high-grade glioma patients or if they discussed cancer-directed therapy with palliative intent. A total of 175 studies were considered for scientific merit and relevance to the aims of this review. The final number of included articles by topic was as follows: Symptoms, functional status, health-related quality of life: 10; caregiver needs: 11; advance care planning: 11; end of life: 11; utilization of primary and specialty palliative care: 10.

### 2.1. Supportive Care Needs of HGG Patients: Symptoms, Functional Impairments, and Distress

HGG has a unique clinical course that differs from that of other advanced cancers in including early cognitive and functional decline, seizures and neurologic deficits, and facing the end of life almost from the time of diagnosis; all of these aspects have a disproportionately negative impact on quality of life (Table 1). [14].

Physical symptoms in HGG are mostly neurologic in nature. In a review of 32 studies, 25 common symptoms were identified in glioblastoma patients, with the following being most prevalent: seizures (37%), cognitive deficits (36%), drowsiness (35%), dysphagia (30%), headache (27%), confusion (27%), aphasia (24%), motor deficits (21%), fatigue (20%) and dyspnea (20%). [15] There is variability within the disease trajectory, with more headache and dizziness at the time of diagnosis, higher rates of treatment side effects during the follow-up phase, and more drowsiness, fatigue, and neurologic deficits in the end-of-life phase. Symptom burden increases during active HGG treatment and hospitalizations, and there is a phase of significant and progressive worsening at the end of life. [14,16,17].

Emotional distress is also common: a systematic review of 42 articles measuring depression in glioma patients revealed a frequency of 15% in studies using clinician-rated measures and 27% in those using patient-reported measures. [18] While both numbers suggest that only a minority of HGG patients experience clinical depression, diagnosis may be challenging given the prevalence of other neurocognitive symptoms. A separate review of 16 qualitative studies provided a more detailed exploration of the emotional consequences of HGG, including shock, disbelief, and powerlessness in the face of the diagnosis; distressing changes in interpersonal relationships due to neurocognitive symptoms, personality and behavior changes; increasing dependence on caregivers; and existential anxiety in the face of a terminal diagnosis. [19] Patients also reported significantly higher illness intrusiveness (the extent to which illness interferes with daily life) than patients with other cancers, including lung and breast, arising in part from the significant decline in physical and cognitive function associated with HGG [20]. 

Indeed, deficits that affect functional status, such as aphasia, vision loss, and motor deficits, appear in nearly half of patients [21]. In a retrospective study of 544 functionally independent patients undergoing resection of glioblastoma, 56% had lost functional independence by 10 months after surgery, increasing to 70% by 18 months [22]. Functional decline that renders the patient difficult to care for at home is a common reason for hospitalization, which may be burdensome for patients, families, and the health care system [23,24]. Moreover, the level of dependence due to physical function is inversely correlated with patient-reported wellbeing and survival [22,25]. 

Like functional decline, cognitive dysfunction is common and affects daily functioning, independence, and medical decision-making [26]. In addition to cognitive impairment caused by the tumor itself, some cancer-directed therapies (surgery and radiation) and symptomatic treatments (anti-epileptic drugs and corticosteroids) have been associated with worsening cognitive deficits [26]. In the last weeks of life, up to 50% of patients have lost their ability to make decisions for themselves, emphasizing the importance of early goals of care discussions [27]. 

Taken together, these factors result in decreased health-related quality of life (HRQOL), an important patient-related outcome measure in brain tumor research [21]. Patients with HGG have lower scores than the general population both in terms of overall quality of life (76.5 vs 85.9 on the FACT-G scale in a sample of 116 Australian patients) and with respect to physical (20.85 vs 25.1), functional (15.6 vs 20.3), and emotional (16.8 vs 21.2) domains [25]. Its significance is underscored by findings from a study of 79 patients with glioma (both low- and high-grade) that 79% of patients with HGG prioritized quality of life over survival when making medical decisions [21]. 

### 2.2. Caregiver Needs

The detrimental effects of HGG extend to caregivers as well [28,29]. The success of caregiver coping strategies may be associated with the patient’s quality of life as well as their own, such that support of the caregiver is also support of the patient [30]. In a systematic review of 35 studies, Applebaum et al. identified the following negative impacts reported by caregivers: loss of identity and changing relationships, isolation, responsibility and guilt, and anxiety about the patient’s death [29]. Because of the early loss of independence described above, caregivers quickly become responsible for assisting the patient with activities of daily living and handling the logistics of medical care, finances, and the home, often while simultaneously dealing with personality and behavior changes in the patient [31,32,33]. This leads to significant distress, as seen in a cohort of Australian caregivers of HGG patients evaluated at the time of the patient’s first chemo-radiotherapy and 3 and 6 months later, where self-rated distress was consistently moderate or high in 60% of caregivers [34]. Evidence of psychological stress was also present in approximately 40% of caregivers at all three time points. Even among caregivers of HGG patients who survive beyond two years from diagnosis, 28% report moderate to severe distress. [35] Specific unmet needs of caregivers have also been detailed and evaluated longitudinally [36]. Key categories of unmet need reported by at least 20% of caregivers at each study time point included access to services (such as accessible parking at the hospital) and advance care planning (such as discussing prognosis or anticipated physical needs and working through feelings about death and dying).

As functional status worsens at the end of life, caregivers may lose the ability to meet the patient’s needs fully without outside assistance, such as from a hospice organization [37]. In a randomized controlled trial of psychological support including cognitive behavioral therapy for 56 caregivers of patients with HGG, the intervention was associated with improved feelings of caregiver mastery (confidence in caregiving abilities), suggesting that some of the burden associated with caregiving in HGG can be alleviated [38]. These findings highlight the importance of incorporating caregivers into palliative care interventions for HGG, as well as the importance of early advance care planning.

### 2.3. Advance Care Planning

Advance care planning is an open, supported discussion between health care providers, patients, and caregivers about the patient’s goals of care. It is especially important for patients with HGG, where the prognosis is invariably poor, and patients often lose their ability to communicate their wishes early in the disease course.

A 2016 systematic review of advance care planning for primary malignant brain tumors revealed highly variable methodology across studies, making it difficult to paint an accurate picture of current advance care planning practices [39]. Only two of the included studies specifically addressed advance care planning (as opposed to advance directives) [27,40]. In one study of a cohort of 155 HGG decedents, 72% had had at least one end-of-life decision made (e.g., withdrawal of dexamethasone, cancer-directed treatment, or artificial nutrition/hydration) [27]. However, 50% of patients lacked decision-making capacity at the end of life, only 40% had an advance directive, and 40% had not discussed end-of-life preferences with their physician. In the other study, among patients with HGG admitted to the neurology or neurosurgery service at a single institution over a 6-month period, 88% had a documented hospice discussion, but at a median of 28 days prior to death [40]. Details of the discussions and subsequent rates of hospice referral were not reported. Sixty-five percent had a do-not-resuscitate order, instituted a median of 31 days before death, suggesting that most patients did not seek non-beneficial interventions at the end of life. Two interventional studies were included in the systematic review, one evaluating the effects of a video decision support tool [41] and one a pilot of comprehensive palliative care [42]. These interventions were associated with a preference for comfort-focused care and with decreased hospital readmission and ICU utilization, suggesting that palliative care interventions can help increase goal-concordant care in this population.

The review further identified a wide range of advance directive completion across studies, with health care proxy documentation rates ranging from 8% to 77% [39]. One retrospective, single-center study of 117 glioblastoma patients aimed to determine adherence to five ASCO palliative care quality measures, including completion of an advance directive (e.g., health care proxy or durable power of attorney documents, living wills, or Medical Orders for Life-Sustaining Treatment), by the third oncology appointment [43]. Fifty-two percent of patients met this goal, though the advance directive in 49% of those cases was a health care proxy alone, with no information about treatment preferences. The low rate of advance care planning in these studies—and the fact that it seems to happen late in the disease course when it does occur—demonstrates substantial room for improvement. This is likely the result of many factors: a 2018 study involving semi-structured interviews with 15 neuro-oncology providers identified emotional difficulty, time (both amount of time available and the “right time”), and lack of clearly defined roles (who will do the planning) as contributors to avoidance of advance care planning [44]. Another theme was uncertainty about what exactly defines advance care planning and how to do it. One study in Australia found that a group of 35 interdisciplinary health care providers recognized limitations in the supportive care available to patients, while also acknowledging the difficult balance between sharing realistic prognostic information and maintaining hope in HGG [45]. 

Along with identifying surrogate decision-makers and delineating treatment preferences, discussion of prognosis is an important aspect of advance care planning. A study of prognostic awareness, defined as awareness of incurability and accurate estimate of life expectancy, was conducted among 50 malignant glioma patients and 32 paired caregivers. Only 40% of patients and 69% of caregivers had full prognostic awareness, meaning they knew the disease was incurable and could estimate their own life expectancy [46]. Twenty percent of patients had no prognostic awareness, meaning they believed high-grade glioma to be a curable disease, and 40% had limited prognostic awareness, meaning they were aware that it was incurable but overestimated life expectancy. Notably, the proportion of patients with limited or no prognostic awareness remained at nearly 60% in patients with multiply recurrent HGG. Caregivers were more aware, with 69% having full prognostic awareness and only 3% having no prognostic awareness. A separate qualitative study also found that caregivers had a more vivid recollection of the initial delivery of the diagnosis and prognosis, which they described as a traumatic experience [47]. Nevertheless, 60% of patients and 72% of caregivers in the prognostic awareness study reported that prognostic information was extremely or very important, and 42% of patients and 50% of caregivers desired more prognostic information [46]. Study authors suggest that the discrepancy in prognostic awareness between patients and caregivers may be due to differences in understanding prognostic information that has been shared, requests from caregivers to receive prognostic information independently, or greater tendency among caregivers to seek prognostic information elsewhere (e.g., the internet). We conclude from the above findings that patients and caregivers do welcome advance care planning. One intervention that looked at ways to improve such communication in this population was the recent Information, Coordination, Preparation, and Emotional (I-CoPE) pilot study of structured, supportive care for HGG, including staged information sharing at the time of diagnosis, following hospital discharge prior to initiation of chemo/radiotherapy, and following completion of radiotherapy [48]. Along with distress screening, emotional support, and care coordination from an I-CoPE care coordinator, this intervention was feasible and acceptable to caregivers, providing a potential template for structured advance care planning interventions. Additional research is needed to define the optimal way to introduce and document advance care planning in this population to improve goal-concordant care at the end of life.

### 2.4. End of Life

The final days and weeks of life present special challenges in HGG (Table 2) [23,49,50]. The symptom profile evolves, with fatigue, drowsiness, and neurologic deficits dominating [16]. Up to 60% of patients are described by their caregivers as “severely disabled” at the end of life [49]. Factors associated with poor quality of life during this period include disability, high levels of stress at the time of diagnosis, and moderate or marked cognitive or personality changes. “Quality of death” may also be suboptimal: A survey conducted in the Netherlands of 81 caregivers of patients with HGG who had died revealed that 25% of caregivers perceived that the patients had died without dignity (as measured on a 5—point Likert scale ranging from “very undignified” to “very dignified) [51]. Factors associated with positive perceptions of the quality of care in the final 3 months of life, according to a survey of 207 family caregivers of HGG decedents in the Netherlands, Austria, and Scotland, including dying in the preferred location, effective treatment of physical symptoms, and satisfaction with information provided. [52].

Despite evidence that palliative care issues are important to HGG patients at the end of life, extant studies suggest they are not prioritized (Table 3 and Table 4). While care patterns vary between countries, hospitalization late in the disease course is common, but not necessarily beneficial [24,52,53,54,55]. For example, in a case-control study of 385 glioblastoma patients at a single center in the United States, 42% of patients were hospitalized within a month of death, and of these hospitalizations, 85% were for management of neurologic decline, which is an expected and frequently irreversible outcome for many patients [24]. Among those hospitalized within a month of death, 32% died in the hospital. Thirty-four percent received intensive care, 11% underwent mechanical ventilation, and 2 patients received cardiopulmonary resuscitation. The study’s authors propose that many of these admissions could have been avoided with early and comprehensive end-of-life discussions. The fact that most study participants chose not to be resuscitated also suggests that most patients do not choose non-beneficial treatments, implying that communicating expected outcomes for a given therapy is essential. Similarly, in an Australian study of 678 HGG patients who survived beyond 120 days from diagnosis, 54% presented to the ED at least once in the 120 days prior to death, spending a median of 11 days in the hospital during that period. The proportion of patients who received inpatient palliative care consultation did increase throughout the disease course, from 5% during the diagnosis admission, to 26% in the final 120 days of life, to 63% during the admission in which the patient died [53]. Compared to patients who did not receive palliative care services, patients who received palliative care during the 120-day period prior to death were 70% more likely to die outside of the hospital (*p* = 0.03). The same authors also compared patients with HGG who died during the diagnosis admission to those who died within 120 days of diagnosis [55]. Patients in the former group were older and more likely to have a multifocal tumor or other medical comorbidities and were less likely to have surgical resection of their tumor. They were also more likely to present through the emergency department or be admitted to the intensive care unit (ICU). These findings suggest that palliative care consultation is pursued only when patients are recognized as close to death, and thus may miss out on the benefits of early palliative care referral, including death outside the hospital and timely hospice enrollment.

According to a retrospective analysis of Medicare claims data, 63% of HGG patients in the United States were enrolled in hospice between 2002 and 2012 [56]. Of those 63%, 11% were enrolled within 3 days of death, and 22% were enrolled within 7 days of death; the median time on hospice was 21 days. In general, patients with malignant brain tumors are referred to hospice less frequently and later in their disease course than other solid tumor patients, and late enrollees tend to have more severe neurologic debilitation than patients referred earlier, suggesting that they do not derive maximal benefit from the service [57,58]. Earlier incorporation of palliative care principles, such as advance care planning, may help identify the most appropriate time for hospice referral on an individual basis.

### 2.5. Primary and Specialty Palliative Care for High-Grade Glioma

**Figure 1:**
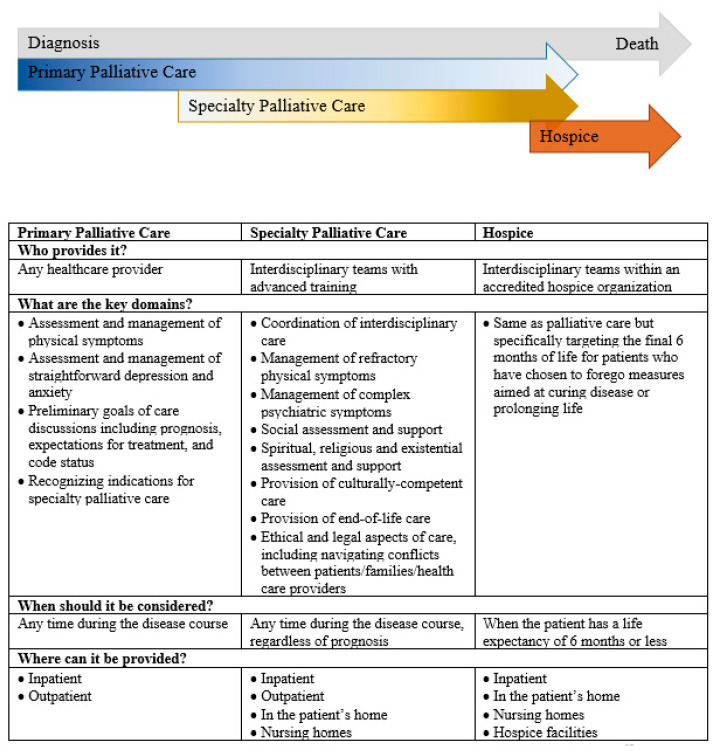
Key differences between primary palliative care, specialty palliative care, and hospice [59,67].

A key unanswered question is who should address the palliative care needs of patients with HGG. Palliative care is divided into two categories: primary palliative care, which can be delivered by any medical provider, and specialty palliative care, which is delivered by an interdisciplinary team of physicians, nurses, social workers, and chaplains with advanced training in palliative care (Figure 1) [63]. Primary palliative care is provided longitudinally by neuro-oncologists and other providers involved in cancer treatment (including neurosurgeons, radiation oncologists, nurses, and social workers); it consists of managing physical and emotional symptoms, as well as preliminary goals of care discussions, including communication about prognosis, expectations for treatment, and code status. Specialty palliative care builds on this in cases of refractory symptoms, complex goals of care discussions, and when needed, end-of-life care and transitions to hospice. Hospice shares the palliative care philosophy of focusing on the quality of life and relief of suffering but is exclusively reserved for the final six months of life. We focus here on primary and specialty palliative care.

The elements of high-quality primary palliative care for patients with advanced cancer are described in a joint statement from ASCO and the American Academy of Hospice and Palliative Medicine (AAHPM) [64]. The statement addresses nine practice domains, including symptom assessment and management, communication about treatment options and limitations on treatment, and end-of-life care as essential skills for all clinicians. While expert opinion among neuro-oncologists supports the joint statement [65,66], there is limited literature regarding primary palliative care interventions for any brain tumor population. One example of comprehensive primary palliative care in neuro-oncology is a home-based program in Italy that enrolled 848 patients with brain tumors (80% HGG) from 2000–2012 [42]. Patients received in-home support, including end-of-life care as needed, from a neurologist-led multidisciplinary team from the time of enrollment until death. Of 529 decedents, 323 (61%) died at home, 116 (22%) died in the hospital, and 90 (17%) died in a hospice facility. Documented end-of-life decisions among those who died at home included steroid withdrawal (45%), delivery of mild hydration (87%), tube feeding (13%), and palliative sedation (11%), though the authors did not describe the decision-making process. Caregivers reported being satisfied with the program on all metrics: home assistance (reported by 98%), nursing (95%), communication (93%), rehabilitation (92%), and social work (88%). The same group separately analyzed 122 patients with glioblastoma who were enrolled in the same program throughout 2012–2013, with similar findings regarding end-of-life care and caregiver satisfaction with care [59]. While these observations are promising, this type of program is resource-intensive and thus difficult to generalize across practice environments. Additionally, palliative care education in neuro-oncology has not been standardized, and not all neuro-oncology providers will feel comfortable addressing care domains such as advance care planning and end-of-life care.

A 2018 survey of 17 (out of 26) neuro-oncology fellowship program directors in the United States found that palliative care curricula are varied and sometimes limited [61]. Two programs reported no formal curriculum; of the 15 that did have formal palliative medicine didactics, most used lectures and readings, while also noting that these are the least effective learning formats. Rotating with a palliative care service was rated most effective but was required in only 2 of the 17 programs. Communication and prognostication were the most commonly identified areas of importance in palliative care education. Time constraints and availability of faculty were identified as the most common obstacles.

For clinicians seeking to provide evidence-based primary palliative care to patients with HGG, the European Association for Neuro-Oncology published guidelines for palliative care in adults with glioma in 2017, based on both evidence and expert opinion [13]. Notably, they concluded that the quality of published evidence in this area is overall low. The 2018 book, “Neuropalliative Care: A Guide to Improving the Lives of Patients and Families Affected by Neurologic Disease,” also provides a useful review of palliative care principles for a neurology audience, including a chapter on malignant brain tumors [66]. There are opportunities for advanced training in communication skills through the Communication Skills Pathfinder, a web portal for connecting clinicians to online modules and in-person training [67]. We propose that adding these skills to their expertise in neuro-oncology may address many of the unmet communication needs described in the literature on HGG, though HGG patients may also require specialty palliative care input to achieve optimal benefit.

### 2.6. Specialty Palliative Care

The eight domains of high-quality specialty palliative care are summarized in Figure 1 [68]. A key benefit of specialty-level care is the addition of the interdisciplinary team of physicians, nurses, social workers, and chaplains with advanced certification and training in caring for patients and families affected by serious illness [69]. Physician and nurse training covers advanced symptom assessment and management, establishing rapport with seriously ill patients and their families, breaking bad news, communicating prognosis, and discussing end-of-life preferences. Palliative care social workers are familiar with community resources for patients and caregivers, and also have expertise in advance care planning, providing bereavement support (including to children), and providing support to patients with a history of trauma or mental illness [70]. Chaplains and other spiritual care providers play an important role in supporting patients and caregivers, and spiritual care interventions such as life review and dignity therapy are associated with reduced levels of the existential distress that accompanies serious illness [71]. All interdisciplinary team members work together to recognize and respond to the needs of seriously ill patients.

Clinical trials of specialty palliative care in other types of advanced cancer have demonstrated beneficial effects on quality of life and satisfaction for patients and caregivers, as well as a reduction in health care utilization and costs without reducing life expectancy [7,8,9,10]. Additionally, palliative care referral is associated with increased and earlier hospice enrollment at the end of life in other cancers.^11^ Specialty palliative care should be considered in cases of refractory symptoms, complex grief or existential distress, interpersonal conflict (between patients, caregivers, and/or health care providers), or requests for non-beneficial treatments [63]. Specific indications for specialty palliative care in HGG are less clearly defined [13]. Few studies have been published specifically addressing the role of specialty palliative care in this unique population, and no randomized, controlled trials have yet been conducted [60,72]. In one observational study of 50 patients with glioblastoma admitted to a tertiary hospital in Australia who had contact with specialty palliative care during that admission, the most common reason for palliative care consultation was “complex discharge planning” (78%), followed by request from a community palliative care service (40%) or symptom management (28%) [72]. The median time from glioblastoma diagnosis to contact with inpatient palliative care was 111 days and the median days from referral to death were 33 days. Five patients (10%) died during the hospitalization, and 18 (36%) were transferred to the palliative care unit, further supporting the idea that referrals are initiated when patients are recognized as being close to death. Potential barriers to increased palliative care utilization for neuro-oncology patients are discussed below.

### 2.7. Challenges in Integrating Palliative Care for Neuro-Oncology

**Figure 2:**
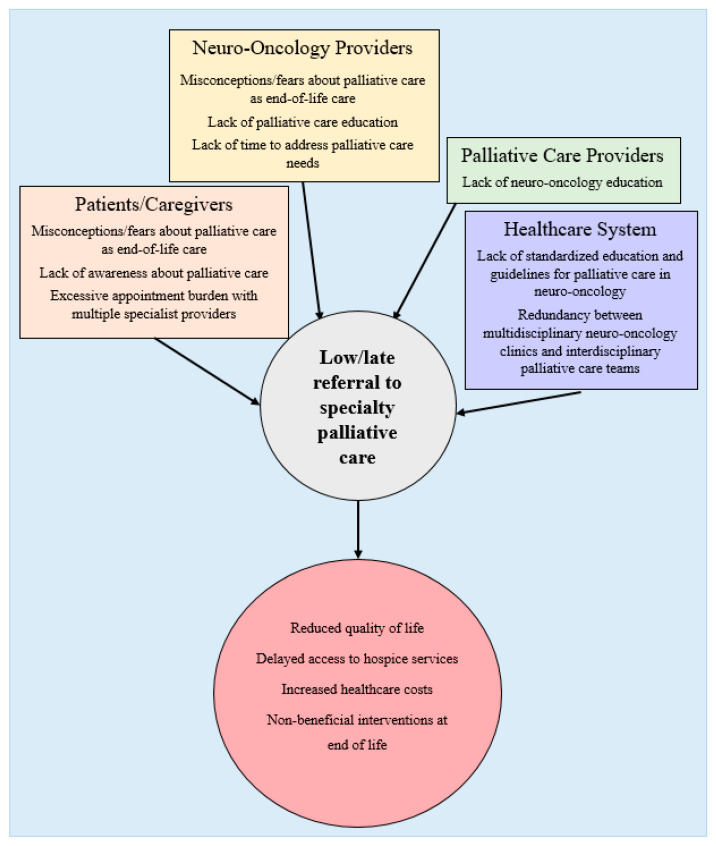
Our novel conceptual model of potential barriers to specialty palliative care referral among patients, providers, and the health care system, based on the literature [77].

There is currently no strong evidence base or expert consensus on the ideal model for integrating palliative care into neuro-oncology, which remains the subject of active research [73,74]. Existing models for outpatient palliative care include oncologists providing primary palliative care without specialty input; oncologists referring to specialty palliative care based on clinical judgment, and integrated models with oncologists and palliative care specialists working as a coordinated team, perhaps co-located in a single multidisciplinary clinic [75,76]. Barriers at the patient, provider, and health systems levels that may limit primary palliative care measures and specialty palliative care referral in the HGG treatment course are illustrated in Figure 2 and Table 5. [77].

With respect to patient perspectives, there may be a lack of knowledge about palliative care and a misconception equating it to hospice and end-of-life care. Semi-structured interviews with patients with primary malignant brain tumors revealed apprehension about palliative care being focused on the time of active dying and the potential of diminishing optimism about disease-modifying treatment [78]. However, they were open to palliative care as emotional support and wished to be educated about it as an option early in their illness. Additional themes included a preference for home-based palliative care to facilitate increased time with family and caregivers (suggesting the burden of additional outpatient appointments as another patient-level barrier), as well as dissatisfaction with brief interactions with providers that did not allow time to discuss the full extent of the family’s worries and hopes. These findings are consistent with another qualitative study in which HGG patients reported feeling that health care professionals were not open to talking about the future and were uncomfortable talking about palliative care, which the patients felt was a barrier in their relationships with those providers [62]. In an observational study of 79 patients with glioblastoma (54 patients; 68%) or brain metastases (25 patients; 32%) who were offered palliative care consultation within 2 months of diagnosis (or diagnosis of relapse), 38% opted to pursue the consultation [60]. Educating providers about appropriate ways to introduce palliative care may help to allay patient fears, and focusing on primary palliative care interventions or integrated models of care may be optimal in reducing appointment burden.

As the central provider managing the care of patients with HGG, neuro-oncologists’ opinions and perspectives about palliative care are likely to impact both their practice of primary palliative care and their referrals to specialty palliative care (Table 4). A 2016 survey of 239 neuro-oncology providers, including neurosurgeons, medical neuro-oncologists, and advanced practice providers, explored their perceptions of specialty palliative care [79]. The majority (97% of respondents) reported having referred at least one patient to specialty palliative care, with 33% referring at least half of their patients. Symptom management was the most common reason for referral (57%), followed by end-of-life care (18%), recurrent disease (10%), and new diagnosis (4%). Thirty-two percent of respondents reported a patient preference for ongoing treatment as a barrier to referral, suggesting they also viewed palliative care as incompatible with the continuation of cancer-directed therapies. This is indeed not the case, as palliative care can be offered along with efforts to treat HGG, including clinical trials of new therapies. Of note, 57% of respondents had some form of palliative care training, thus palliative care utilization may be higher in this group than among neuro-oncology providers generally. Overall, the literature reviewed here suggests that additional training for neuro-oncologists would enhance both the practice of primary palliative care and the implementation of specialty palliative care when appropriate.

Similarly, palliative care specialists may benefit from increased education on neurologic disease, including high-grade glioma and other brain tumors. Although AAHPM’s core competencies for palliative medicine trainees include performing detailed neurologic exams, managing neuropsychiatric disorders, and recognizing indications for specialty neurology referral, there are no guidelines or studies of a neurology curriculum [80]. Most palliative care specialists have an internal or family medicine background, and as of 2014, there were only 110 physicians who were board-certified in both psychiatry or neurology and palliative medicine in the United States [81]. Additional education would likely be beneficial both in caring for patients with HGG and in gaining the confidence of referring neuro-oncologists. In some cases, patients will receive all their care from providers other than their neuro-oncologist at the end of life, and augmenting neurology education for palliative care and hospice providers may improve transitions of care in that context [82]. 

### 2.8. Future Directions

The existing literature, as reviewed above, provides ample evidence of the palliative care needs of patients with HGG. Recently, several expert recommendations and guidelines have been published addressing the need to provide palliative care in this population [13,75,83]. However, questions remain about the optimal timing and model of palliative care delivery to improve quality of care and patients’ and caregivers’ experience. Regarding timing, we propose that it is necessary to re-focus research efforts away from the end of life, given that patients are likely to derive the most benefit before reaching a crisis phase, while they are still able to communicate their goals and treatment preferences. Research on barriers to primary and specialty palliative care services at key time points in the disease trajectory is also needed. Future studies should build on what is already known about the perspectives of patients, caregivers, and providers (both neuro-oncologists and palliative care specialists) about appropriate models of providing palliative care. These studies will lay the groundwork for clinical trials of palliative care interventions in HGG to determine the appropriate balance of primary palliative care as provided by neuro-oncologists and specialty palliative care via consultation or co-management. Ultimately, additional methodologically sound studies of palliative care for HGG are needed to support practice changes.

## 3. Conclusions

High-grade glioma is a devastating diagnosis with profoundly negative effects on quality of life for patients and their caregivers, along with poor prognosis that makes early advance care planning an imperative. Palliative care has the potential to alleviate the suffering of these patients when provided simultaneously with standard treatment of their cancer, yet it seems to be under-utilized in HGG, for a variety of reasons including complex communication dynamics, limited provider training, and a paucity of research into effective primary and specialty palliative care interventions. Further study is needed to evaluate barriers to early palliative care in this population and develop integrative models of care that meet the needs of patients, caregivers, and neuro-oncologists alike.

## Figures and Tables

**Table 1:** Summary of the literature on the supportive care needs of patients with HGG throughout the disease trajectory. Articles that fall into multiple categories are included only once.

Author (Year)	Country	Number of Centers	Study Type	Number of Participants	Key Findings
**Physical and Emotional Symptoms**					
**Ijzerman-Korevaar (2018) [15]**	N/A	N/A	Systematic review	32 studies addressing symptoms, side effects, and adverse events in glioma patients	• Identifies 10 most common symptoms in different phases of glioma trajectory
**Psychological Distress**					
**Rooney (2013) [18]**	Scotland	2	Prospective cohort	154 patients with glioma (low or high grade)	One-third of patients reported significant emotional distress Patients with high distress early in disease
**Sterckx (2013) [19]**	N/A	N/A	Systematic review	16 qualitative studies of impact of HGG on everyday life	• Sources of distress include death anxiety, loss of autonomy, and behavior/personality changes
**Edelstein (2015) [20]**	Canada	1	Cross-sectional survey focusing on psychiatric components of care	73 patients with GBM	GBM patients have a less positive affect, more depression, more illness intrusiveness than other cancer patients High cancer symptom burden associated with illness intrusiveness and depression
**Functional Status**					
**Gabel (2019) [21]**	USA	1	Retrospective analysis	58 patients with HGG and 21 with LGG	Physical impairment was present in 28.6% Cognitive impairment was present in 43.9%
**Chaichana (2011) [22]**	USA	1	Retrospective analysis	544 patients with KPS ≥ 80 ^a^ who underwent first-time resection of primary or secondary GBM	56% of patients were no longer functionally independent at 10 months post-op Older age, comorbid CAD, COPD, HTN, pre- or postoperative motor or language deficits predictors of losing functional independence
**Cognitive Dysfunction**					
**Bergo (2019) [26]**	N/A	N/A	Narrative review	Studies addressing cognition and HRQOL in HGG	Cognitive deficits are common and may be caused by HGG and by treatment (surgery, radiation, AEDs, corticosteroids) Cognitive rehabilitation may have some benefit
**Sizoo (2012) [27]**	Netherlands	3	Cross-sectional survey	Physicians and relatives of 155 deceased HGG patients	>50% of patients lacked capacity for decision-making at the end of life 40% of physicians did not discuss end-of-life preferences with patients
**Health-Related Quality of Life**					
**Gabel (2019) [21]**	USA	1	Retrospective analysis	58 patients with HGG and 21 with LGG	• Majority of patients in both groups prioritized HRQOL over survival
**Halkett (2015) [25]**	Australia	4	Prospective cohort	116 HGG patients	Poor HRQOL, high distress, high unmet needs when commencing radiotherapy Low education and financial resources associated with lower HRQOL

^a^ Karnofsky Performance Status (KPS) is a tool for providers to describe a patient’s functional status, ranging from 100 (no complaints, no evidence of disease) to 0 (dead). PBT: Primary brain tumor; GPC: General palliative care; HRQOL: Health-related quality of life; GBM: Glioblastoma; WHO: World Health Organization; HGG: High-grade glioma; LGG: Low-grade glioma; CAD: Coronary artery disease; COPD: Chronic obstructive pulmonary disease; HTN: Hypertension; AED: Anti-epileptic drug.

**Table 2:** Summary of the literature on end-of-life supportive care needs and health care utilization in HGG.

Author (Year)	Country	Number of Centers	Study Type	Number of Participants	Key Findings
**Thier (2016) [16]**	Austria	1	Retrospective analysis	57 patients with GBM	• Identifies most common symptoms and medications in last 10 days of life
**Sizoo (2010) [17]**	Netherlands	1	Retrospective analysis	55 patients with HGG	• Depressed mental status, dysphagia were most common symptoms in final week of life
**Oberndorfer (2008) [23]**	Austria	1	Retrospective chart review	29 patients with GBM	Health care utilization (medications, diagnostic tests, procedures) increased with proximity to death Mean time from end of cancer-directed therapy to death was 10 weeks
**Diamond (2017) [24]**	USA	1	Retrospective data analysis	385 GBM patients	42.6% of patients were admitted within 30 days of death 34% of admitted patients had ICU-level care 13% had mechanical ventilation, 1% had CPR
**Sizoo (2014) [27]**	N/A	N/A	Systematic Review	17 studies addressing the end-of-life phase for HGG patients	Symptom burden at the end of life is high and burdensome for patients and caregivers Functional and cognitive decline are significant issues HGG patients have ACP later in their disease course than other patients with neurologic disease
**Koekkoek (2016) [50]**	N/A	N/A	Narrative Review	N/A	Summarizes literature on end-of-life symptom management in HGG Recommends early ACP Reviews caregiver needs
**Sizoo (2012) [51]**	Netherlands	3	Cross-sectional survey	101 providers 50 relatives	25% of caregivers viewed patients as having died without dignity Goals of care discussions were reported as limited Preserved communication, positive relationships with health care providers, and lack of health care transitions were associated with dignified death
**Koekkoek (2014) [52]**	Netherlands Austria Scotland	7	Cross-sectional survey	207 caregivers of HGG decedents	• Predictors caregiver satisfaction with end-of-life care include dying in preferred location; symptom control; meeting of informational needs
**Sundararajan (2014) [53]**	Australia	Many	Retrospective cohort	678 malignant glioma patients	26% of patients died outside the hospital 49% died in a palliative care/hospice setting 25% died in an acute hospital bed
**Alturki (2014) [54]**	Canada	Many	Retrospective analysis	1623 decedents with primary intracranial tumors	90% of patients were admitted to hospital in last 6mo of life 23% spent ≥3 months in acute care in last 6mo 49% died in hospital, 10% at home, 40% in palliative care facility
**Collins (2014) [55]**	Australia	Many	Retrospective cohort	482 malignant glioma patients who died within 120 days of diagnosis	62% of patients who died during diagnosis admission was admitted to a palliative care bed; 38% died in an acute hospital bed 12% of patients who survived diagnosis admission had a palliative care consultation Presence of cognitive or behavioral symptoms was strongest predictor of death during diagnosis admission (OR 3.1)

GBM: Glioblastoma; HGG: High-grade glioma; ACP: Advance care planning; ICU: Intensive care unit; CPR: Cardiopulmonary resuscitation.

**Table 3:** Summary of the literature on current utilization of primary palliative care, specialty palliative care, and hospice among patients with HGG.

Author	Country	Number of Centers	Study Type	Number of Participants	Key Findings
**Primary Palliative Care**					
**Sizoo (2012) [27]**	Netherlands	3	Cross-sectional survey	Physicians and relatives of 155 deceased HGG patients	• 40% of physicians did not discuss end-of-life preferences with patients
**Gofton (2012) [40]**	USA	1	Retrospective analysis	168 patients with any CNS tumor (101 with HGG)	77% of HGG patients had documented HCP 65% had DNR order 85% had hospice care discussion (68% enrolled in hospice) 12% of patients received specialty palliative care consultation
**El-Jawahri (2010) [41]**	USA	1	Randomized controlled trial of a verbal narrative of end-of-life treatment options vs verbal narrative plus a video depicting the treatments	50 patients with HGG (23 in intervention arm, 27 controls)	In intervention arm, no participants chose life-prolonging care (vs 26% of controls; *p* <0.0001) In intervention arm, 91% chose comfort care (vs 22% of controls; *p* <0.0001) 82.6% of participants reported being ‘very comfortable’ watching the video
**Pace (2014) [42]**	Italy	1	Pilot intervention of in-home neurology visits, neuro-rehabilitation, psychological support, nursing assistance	848 patients with any brain tumor	61% of patients who died did so at home; 22% died in acute hospitals; 17% died in hospice Significant reduction in hospital readmission rates in final 2 months of life compared to controls (16.7% vs 38%, *p* <0.001)
**Hemminger (2017) [43]**	USA	1	Retrospective cohort	117 decedents with GBM	52.1% had any advance directive by the 3^rd^ oncology visit (49.2% health care proxy, 36.1% MOLST, 13.1% living will, 1.6% non-hospital DNR) 26.5% had no advance directive prior to the final month of life 36.8% had a palliative care consult at any point in the disease course
**Pompili (2014) [59]**	Italy	1	Pilot intervention of in-home neurology visits, neuro-rehabilitation, psychological support, nursing assistance	122 patients with GBM	Among 64 decedents, 53.1% died at home; 34.4% died in a hospice facility; 12.5% died at the hospital Caregivers reported satisfaction with home assistance (97%); nursing (95%); communication (90%); rehabilitation (92%); and social work (85%).
**Specialty Palliative Care**					
**Sundararajan (2014) [53]**	Australia	Many	Retrospective cohort	678 malignant glioma patients	Patients with high symptom burden 5x more likely to receive palliative care in hospital Patients who receive palliative care are more likely to die at home
**Collins (2014) [55]**	Australia	4	Retrospective cohort	1160 decedents with PMBT	78% of pts who died during diagnosis admission received a palliative care consult 12% of pts who survived diagnostic admission but died within 120 days received a palliative care consult 5% of patients surviving admission and >120 days received a palliative care consult
**Seekatz (2017) [60]**	Germany	1	Serial cross-sectional survey	54 patients with GBM	38% of patients chose palliative care when offered within 2 months of diagnosis Patients seen by palliative care had greater improvements in pain and distress than those with no palliative care contact
**Hospice**					
**Forst (2017) [56]**	USA	1	Retrospective analysis	12437 decedents with malignant glioma	Predictors of hospice enrollment: Older age, female sex, more education, white race, lower median income 77% of enrollees were on hospice >7 days, 89% >3 days
**Diamond (2016) [57]**	USA	1	Retrospective cohort	160 decedents with PMBT who enrolled in hospice prior to death	23% of decedents enrolled within 7 days of death Late enrollees are often more severely debilitated Risk factors for late referral: Male sex, low socioeconomic status, lack of health care proxy
**Dover (2018) [58]**	USA	1	Retrospective analysis	1323 deceased Medicare beneficiaries with a malignant brain tumor (383 with PMBT, 940 with SMBT)	24% of PMBT patients had late (1–3 days prior to death) or no hospice care Risk factors for late or no referral: Non-white race, male sex, receipt of any hospital-based care in the final 30 days of life Average decrease of $12,138 in Medicare expenditures in hospice enrollees in PMBT

GBM: Glioblastoma; ACP: Advance care planning; MOLST: Medical orders for life-sustaining treatment; DNR: Do not resuscitate; PMBT: Primary malignant brain tumor; SMBT: Secondary malignant brain tumor.

**Table 4:** Summary of the literature on unmet palliative care needs among patients with high-grade glioma.

Author	Country	Number of Centers	Study Type	#Of Participants	Gaps Identified
**Pace (2017) [13]**	N/A	N/A	Systematic Review and Expert Opinion	223 articles on palliative care needs and management of glioma	Overall limited evidence on palliative care delivery for glioma Need to study fatigue, behavioral symptoms, caregiver needs, and ACP
**Halkett (2018) [36]**	Australia	4	Prospective cohort	118 caregivers of HGG patients	Caregivers have high levels of unmet supportive care needs throughout the disease trajectory Over 25% of caregivers reported a lack of information about prognosis as important at all stages
**Sizoo (2012) [27]**	Netherlands	3	Cross-sectional survey	101 providers 50 relatives of decedents with HGG	• Physicians are often unaware of patients’ end-of-life preferences
**Gofton (2012)^40^**	USA	1	Retrospective data analysis	101 deceased HGG patients	15% of patients had no documented end-of-life discussions 23% had no health care proxy 35% had no DNR order
**Hemminger (2017) [43]**	USA	1	Retrospective cohort	117 decedents with GBM	• Patients received late ACP documentation and minimal early palliative care
**Diamond (2017) [46]**	USA	1	Mixed methods (prognostic awareness assessment tool and semi-structured interviews)	50 patients with HGG with 32 matched caregivers	20% of patients had no prognostic awareness 40% of patients had limited prognostic awareness 42% of patients and 50% of caregivers desired more prognostic information
**Sizoo (2014) [49]**	N/A	N/A	Systematic Review	17 studies addressing the end-of-life phase for HGG patients	• Limited research and no adequate guidelines on end of life care for HGG patients, including symptom management, ACP, and organization of care
**Collins (2014) [55]**	Australia	4	Retrospective cohort	1160 decedents with PMBT	• Under-utilization of palliative care in patients who survived a first hospital admission but died within 120 days
**Forst (2017) [56]**	USA	1	Retrospective analysis	12,437 decedents with malignant glioma	• Patients often referred late (<7 days before death) to hospice
**Mehta (2018) [61]**	USA	17	Cross-sectional survey	17 neuro-oncology fellowship program directors	• No consistent palliative care education for neuro-oncology fellows
**Philip (2014) [62]**	Australia	2	Qualitative interviews	10 patients with HGG	• Patients perceived providers as focused on “here and now,” lacking openness about the future, reluctant to discuss palliative care

HGG: High-grade glioma; DNR: Do not resuscitate; GBM: Glioblastoma; ACP: Advance care planning; PMBT: Primary malignant brain tumor.

**Table 5:** Summary of the literature on the view and preferences of high-grade glioma patients and neuro-oncologists with respect to palliative care.

Author	Country	Number of Centers	Study Type	#Of Participants	Key Figurendings
**Patients**					
**Seekatz (2017) [60]**	Germany	1	Serial cross-sectional survey	54 patients with GBM	38% of patients chose palliative care when offered within 2 months of diagnosis Patients seen by palliative care had greater improvements in pain and distress than those with no palliative care contact
**Vierhout (2017) [78]**	Canada	1	Qualitative interviews	39 patients with malignant brain tumor	Patients want palliative care at home; open to palliative care if it does not decrease optimism; prefer to receive palliative care early
**Philip (2014) [62]**	Australia	2	Qualitative interviews	10 patients with HGG	Patients felt health professionals avoided talking about the future, wished they had talked more about palliative care Patients had a high level of uncertainty about what to expect
**Neuro-oncologists**					
**Llewellyn (2017) [44]**	UK	1	Qualitative interviews	15 interdisciplinary health care providers	Providers see ACP as important but engage in it infrequently 3 main factors for avoidance: Time, lack of clarity in whose responsibility it is, uncertainty about what ACP is
**Philip (2015) [45]**	Australia	3	Qualitative interviews	35 interdisciplinary health care providers	Providers see limitations in current provision of supportive care Challenges balancing hope and prognosis More preventive care is needed
**Walbert (2016) [79]**	USA	Many	Cross-sectional survey	239 interdisciplinary neuro-oncology providers	51% of providers uncomfortable treating end of life issues and symptoms 50% prefer “supportive care” to “palliative care” 32% feel palliative care incompatible with cancer-directed therapy Provider level, specialty, gender, training in palliative care and neuro-oncology influence utilization of palliative care and hospice

GBM: Glioblastoma; ACP: Advance care planning.
